# Examining the connection between BRCA1/2 mutations and uterine cancer: a comprehensive review

**DOI:** 10.1097/MS9.0000000000005267

**Published:** 2026-06-19

**Authors:** Safa Mohammed Saleem, Syed Aasim Syed Nasir, Faiza Nida, Muddassir Khalid, Harsh Gandhi, Muhammad Hassaan Javaid, Abhishek Roy

**Affiliations:** a Department of Medicine, Tbilisi State Medical University, Tbilisi, Georgia; b Department of Medicine, East European University, Tbilisi, Georgia; c Department of Medicine, Nishtar Medical University, Multan, Pakistan; d Department of Medicine, Shifa College of Medicine, Islamabad, Pakistan

**Keywords:** BRCA1/2 mutations, endometrial cancer, genetic predisposition, precision oncology, uterine serous carcinoma

## Abstract

DNA fix needs BRCA1 and BRCA2. These genes stop bad cell growth. They sit on links 17 and 13. Bad changes in some genes raise the risk of womb cancer. This is true for a type called serous. These faults also boost the chance for womb and breast bad growth. BRCA1 faults lead to more severe womb tumors than BRCA2 faults. Drugs like tamoxifen increase this risk more. More breast trouble adds risk, too. The risk of womb cancer in a lifetime is still small – not quite 3% for BRCA1 folk and just 1 or 2% for BRCA2 folk. Yet, BRCA1 faults might bring worse sorts of womb growth. These have bad cell traits and weak fix tools. Now, rules do not say all BRCA folk must have their wombs removed or get womb checks. They say to talk hard about risks. Think of drug use, weight, and past breast issues. New tools look at many cell parts at once. This helps grasp how BRCA links to bad growth. It lets us pick just the right cures, like PARP drugs. But past studies differ. We need wide, long studies with many groups of folks. This will show the real womb cancer risk. So, BRCA faults do play a small but key part in a bad womb growth type. But they mainly boost breast and egg trouble.

## Introduction

**Figure 1.**
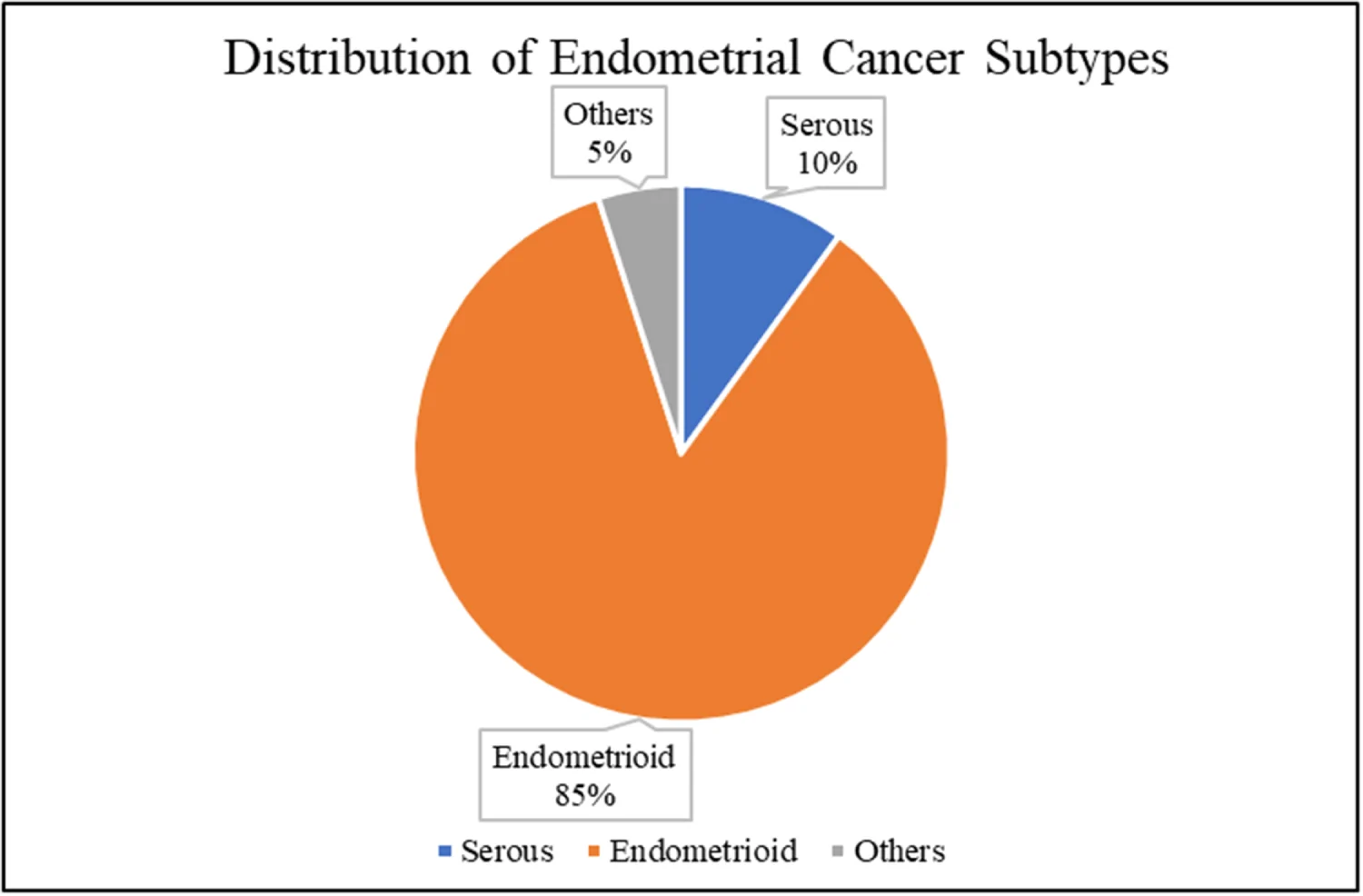
Distribution of endometrial cancer subtypes.

BRCA1 (chromosome 17q21) and BRCA2 (chromosome 13q12.3) are autosomal-dominant tumor-suppressor genes that are required for homologous recombination to fix double-strand breaks in DNA. With an estimated lifetime risk of up to 80% for BRCA1 and 40–60% for BRCA2 carriers for breast cancer, and around 55% (BRCA1) and 25% (BRCA2) for ovarian cancer, germline pathogenic mutations enhance genomic instability, impede DNA damage repair, and increase the risk of malignancies^[^1^]^. Less than 10% of instances of endometrial carcinoma are of the serous (papillary) subtype, but because of its aggressive biology and dismal prognosis, it contributes disproportionately to morbidity and mortality. Their clinical significance is underscored by the fact that these tumors often show TP53 mutations and share genetic similarities with ovarian high-grade serous carcinoma (Fig. 1) ^[^2^]^.

HIGHLIGHTS

- BRCA1 mutations show a stronger link to uterine serous carcinoma than BRCA2.
- Lifetime uterine cancer risk remains low but increases with BRCA1, tamoxifen use, and prior breast cancer.
- Evidence is mixed across studies and populations, with ethnic variation in risk patterns.
- Current guidelines do not advise routine hysterectomy; decisions require individualized counseling.
- Multi-omics and precision oncology approaches may refine risk prediction and targeted therapies.

It was found that three out of 151 uterine serous carcinoma (USC) patients (2.0%) who had their 30 tumor suppressor genes sequenced had germline loss-of-function mutations in BRCA1. The incidence of BRCA1 mutations rose to 9% among 22 women who had a history of breast cancer in addition to USC, indicating a genetic susceptibility in this subgroup^[^3^]^. Seventeen incident endometrial cancers were found in a multinational prospective cohort of 4456 carriers of the BRCA1/2 mutation who were monitored for 5.7 years (13 in BRCA1, four in BRCA2). The standardized incidence ratio (SIR) was substantially higher for BRCA1 carriers than for BRCA2 carriers. The SIR of 4.14 was substantially higher in tamoxifen users than in non-users, at 1.67^[^4^]^.

**Figure 2.**
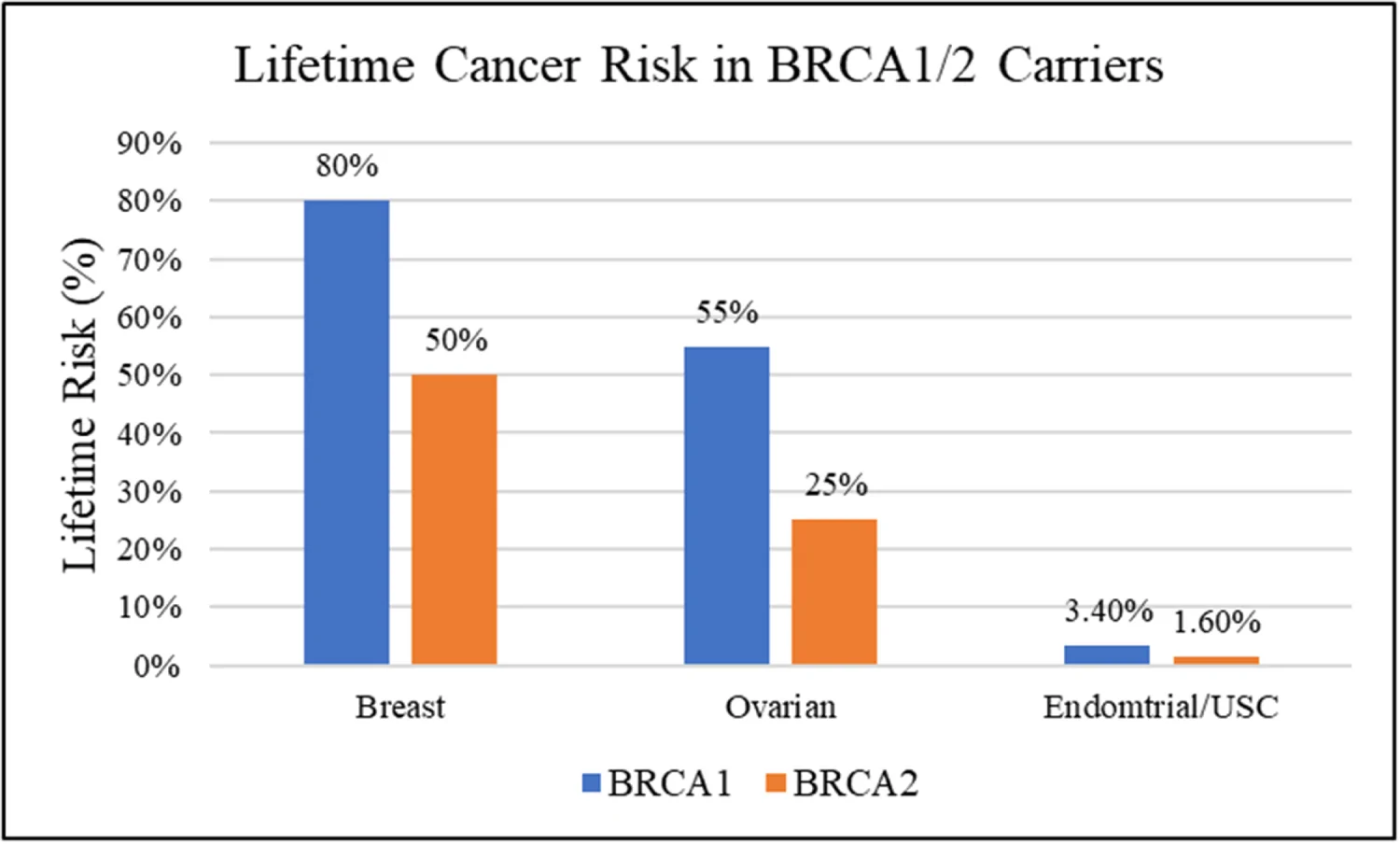
Cancer Risk in BRCA1/2 carriers.

The cumulative incidence from age 40 to 70 was 3.4% for BRCA1 and 1.6% for BRCA2, according to a similar study of 4959 mutation carriers conducted over 6.7 years. Exposure to tamoxifen was associated with a 2.2-fold increased risk. However, a UK-based investigation of 2609 women revealed no discernible rise in the risk of serous-like or total endometrial cancer, and the sequencing of 15 serous endometrial tumors revealed no BRCA harmful mutations (Fig. 2)^[^5^]^. BRCA1/2 mutations are found in about 2% of cases of uterine cancer, according to meta-analyses. Tamoxifen use and previous breast cancer were reported to contradict SIRs of 2–5 for BRCA1, especially in USC^[^6^]^. A different meta-analysis with 13 871 carriers found that the prevalence of USC was 0.16% and that of endometrial cancer was around 0.6%^[^7^]^.

This study addresses the clinical consequences, molecular biology, and epidemiology of BRCA1/2 mutations in connection with uterine cancer. There is still conflicting evidence, despite encouraging correlations. Outlining BRCA1/2 functions, examining the burden of uterine cancer, particularly USC, and critically assessing the genetic relationship and implications for counseling and risk-reducing gynecologic surgery are the objectives of this review.

## Discussion

### BRCA1/2 mutation biology and cancer pathogenesis

BRCA1 and BRCA2 encode proteins that function in the same pathway to maintain genome stability. While BRCA2 primarily facilitates homologous recombination (HR), BRCA1 has broader roles, including DNA repair and activation of cell cycle checkpoints. Mutations in either gene confer similar cancer risks, highlighting their functional linkage, even though the precise interaction between them remains unclear. Together, they help protect the genome from double-strand breaks that occur during DNA replication^[^8^]^.

BRCA1, BARD1, BRCA2, and PAL B2 maintain genome stability by repairing DNA double-strand breaks through HR. Their tumor suppressor function means that mutations increase cancer risk, notably in hereditary breast and ovarian cancer (HBOC) syndrome. Biallelic mutations in BRCA1, BRCA2, and PALB2 (FANCS, FANCD1, FANCN) can also cause Fanconi anemia. Insights into these pathways have enabled targeted therapies, such as PARP inhibitors, which exploit synthetic lethality to selectively eliminate cancer cells deficient in HR^[^9^]^.

BRCA1 and BRCA2 are essential for maintaining genomic stability by repairing DNA double-strand breaks (DSBs) through HR, a high-fidelity repair process. BRCA1 initiates HR by promoting DNA end resection, while BRCA2 recruits and loads RAD51 onto single-stranded DNA to facilitate strand invasion and precise repair. Besides DSB repair, both proteins stabilize stalled replication forks and support the DNA damage tolerance response during replication stress. Mutations in BRCA1/2 disrupt HR, rendering tumor cells highly sensitive to poly (ADP-ribose) polymerase inhibitors (PARPi), which convert single-strand breaks into DSBs. This causes synthetic lethality, selectively killing BRCA-deficient cancer cells. These mechanisms have revolutionized precision oncology by enabling targeted therapies exploiting BRCA deficiencies. Additionally, BRCA1 and BRCA2 interact with various protein complexes that coordinate DNA damage response and genome maintenance, highlighting their critical roles in protecting genomic integrity and ensuring accurate DNA repair^[^10^]^.

Germline mutations in a single copy of BRCA1 or BRCA2 substantially raise the risk of breast and ovarian cancers, with estimated lifetime risks of about 72 and 69% for breast cancer, and 44 and 17% for ovarian cancer, respectively. These genes function as tumor suppressors by maintaining chromosome stability during cell division. Bi-allelic inactivation results in severe cellular defects and heightened sensitivity to DNA damage. According to the “two-hit” model, carriers inherit one defective copy, while the remaining wild-type allele is lost in tumor cells through loss-of-heterozygosity, completely abolishing BRCA function and driving genomic instability that promotes carcinogenesis^[^9^]^.

Hereditary breast and ovarian cancer syndrome (HBOC) is primarily caused by inherited mutations in the BRCA1 and BRCA2 genes, which are crucial for maintaining genomic stability by repairing DNA DSBs through HR. Mutations in these genes significantly increase the likelihood of breast and ovarian cancers, with BRCA2 mutations also linked to higher risks of prostate, pancreatic, and melanoma cancers^[^11^]^. People with HBOC commonly develop early-onset breast cancer, usually before age 50, ovarian cancer (including fallopian tube and primary peritoneal cancers), multiple breast tumors, and male breast cancer. The risk varies depending on whether the mutation is in BRCA1 or BRCA2; BRCA1 mutations are mainly connected to breast and ovarian cancers, while BRCA2 mutations raise the risk for male breast, prostate, and pancreatic cancers^[^11^]^. Diagnosis is made through molecular genetic testing, identifying pathogenic variants in BRCA1 or BRCA2, especially for those with a personal or family history of early breast cancer, ovarian cancer, multiple breast tumors, or male breast cancer. Management includes increased cancer screening, preventive surgeries like bilateral mastectomy and oophorectomy, chemoprevention with drugs like tamoxifen, and targeted therapies such as PARP inhibitors. Genetic counseling is vital for at-risk families. Other hereditary syndromes with similar features include Lynch syndrome, Li-Fraumeni syndrome, and Cowden syndrome, each requiring specific care strategies^[^11^]^.

### Epidemiology and risk of uterine cancer in BRCA1/2 mutation carriers

A multicenter cohort study conducted in the Netherlands involving 5980 women with inherited BRCA1 or BRCA2 mutations examined their risk of developing endometrial cancer (EC). The findings indicated that carriers of BRCA mutations have roughly a 2.8 times higher risk of EC compared to the general population. Women with BRCA1 mutations faced an elevated risk, with a SIR of 3.51 overall, and an even greater risk for aggressive serous-like EC subtypes (SIR 12.64) and TP53-mutated tumors. Meanwhile, BRCA2 mutation carriers showed a comparatively smaller increase in risk (SIR 1.70 overall and 5.11 for serous-like EC). Despite these raised risks, the absolute lifetime risk by age 75 remained relatively low – approximately 3.0% overall and 1.1% for serous-like EC. These results highlight the importance of individualized risk evaluation and counseling for BRCA mutation carriers concerning uterine cancer^[^12^]^. A systematic review and meta-analysis conducted in 2023 examined 21 cohort and two cross-sectional studies, identifying a 2% occurrence of BRCA1/2 mutations among uterine cancer patients. The researchers propose that individuals carrying these mutations may exhibit increased awareness and monitoring for uterine cancers^[^6^]^. A 2020 meta-analysis demonstrated a modestly increased risk of endometrial cancer among BRCA mutation carriers, especially those with BRCA1 mutations, showing a cumulative incidence of 3.4%^[^7^]^.

**Figure 3.**
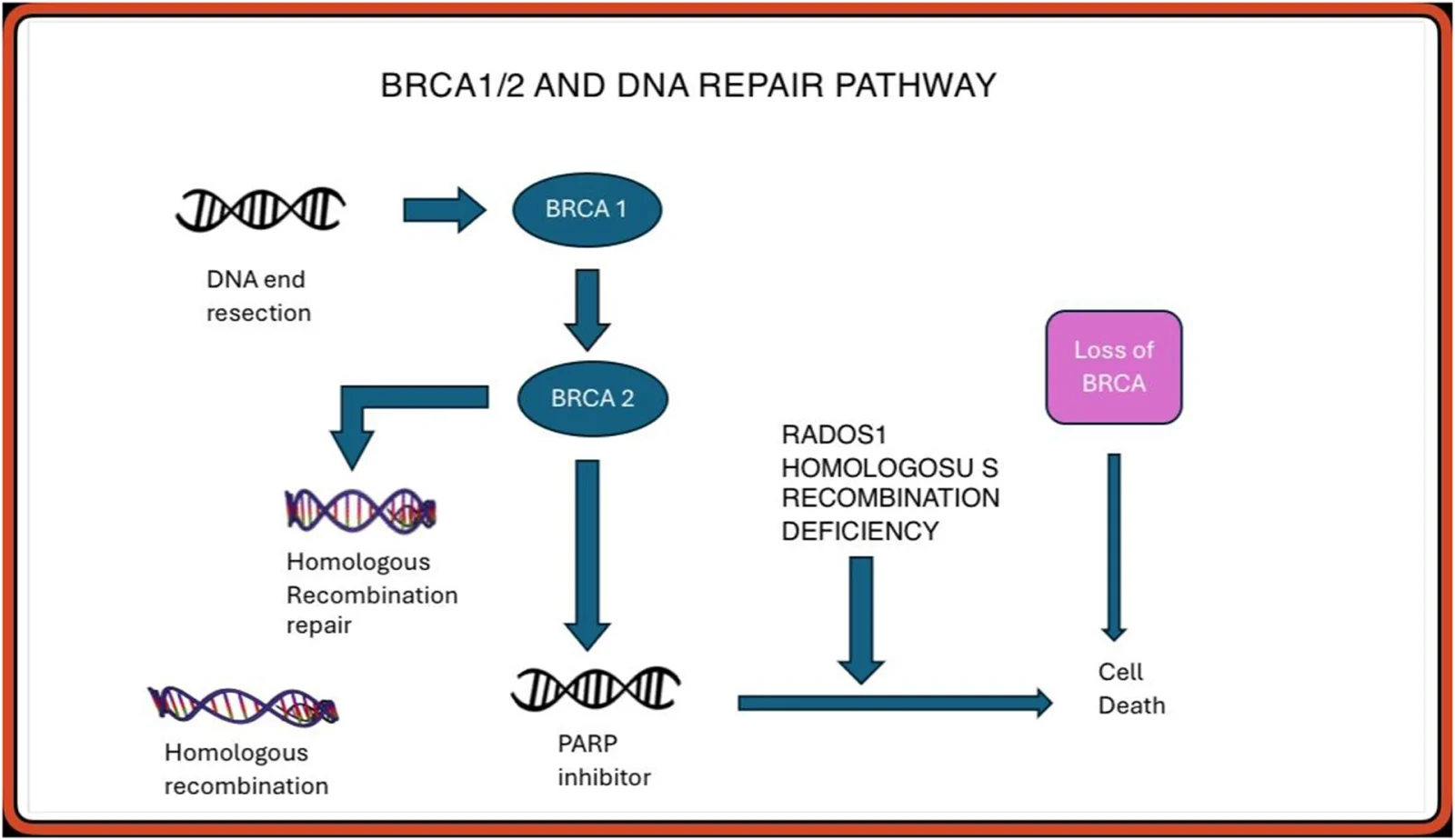
Schematic of BRCA1/2 in DNA repair.

**Figure 4.**
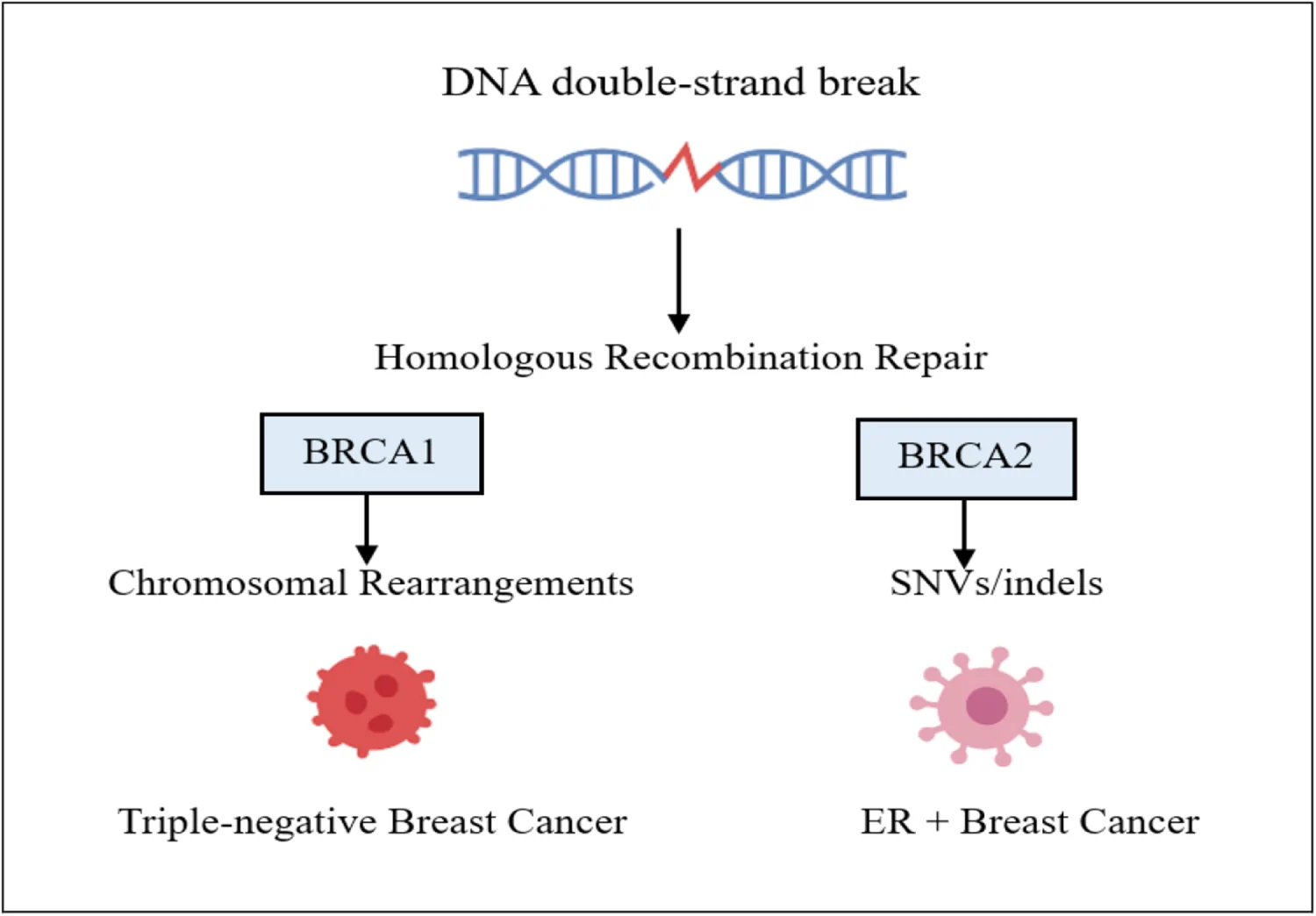
BRCA1/2 and DNA repair pathway.

For HR to repair DNA DSBs, BRCA1 and BRCA2 are necessary. The majority of hereditary ovarian malignancies and almost 10% of all ovarian cancer cases are caused by mutations in these genes, which result in genomic instability, or “BRCAness” (Fig. 3). Despite being frequently taken into account jointly, new research shows that they differ significantly. Carriers of BRCA2 mutations in serous ovarian cancer typically have better treatment outcomes and greater survival rates than those with BRCA1 mutations or no mutations. The unique functions that each protein performs in DNA repair and the higher correlation between BRCA2 mutations and a hypermutator phenotype may be the cause of these variations (Fig. 4). These results are crucial for directing therapy choices and patient care^[^13^]^. People carrying BRCA1 mutations face an approximate 40–45% lifetime risk of ovarian cancer, while BRCA2 mutation carriers have a lower risk of around 10–20%, generally occurring at a later age. BRCA1 is predominantly associated with triple-negative breast cancer, whereas BRCA2 is more frequently linked to estrogen receptor–positive breast cancer. Additionally, individuals with BRCA2 mutations have an elevated likelihood of developing male breast, prostate, and pancreatic cancers^[^14^]^. BRCA1-deficient tumors, especially triple-negative breast cancers, show increased immunoregulatory activity and unique tumor–immune microenvironments, whereas BRCA2-deficient tumors generally lack this immune enrichment. In terms of mutation profiles, BRCA2-deficient tumors exhibit more single-nucleotide variants and small insertions/deletions (indels), potentially enhancing their immunogenicity, while BRCA1-deficient tumors display larger chromosomal rearrangements, which are linked to a poorer response to immunotherapy (Fig. 4)^[^15^]^.

The association between BRCA1/2 mutations and the risk of uterine cancer differs across populations and ethnic groups. Studies show that BRCA1 mutations are linked to a higher risk of uterine cancer, particularly the serous subtype of endometrial cancer, while BRCA2 mutations generally do not confer the same increased risk. These patterns, however, may vary among different ethnic groups due to both genetic and environmental factors^[^16^]^. Studies indicate that people of Ashkenazi Jewish descent have a higher frequency of BRCA1/2 mutations. These distinctive mutation patterns may impact their risk of various cancers, including uterine cancer. Evidence suggests that African American women may have BRCA mutations that are distinct from those found in other racial groups. These genetic differences can influence their risk of uterine cancer and highlight the importance of implementing comprehensive and inclusive genetic screening programs^[^16,17^]^.

### Uterine cancer subtypes and histopathological correlation

Uterine cancers are split into two main types: endometrioid (Type I) and non-endometrioid (Type II). Endometrioid cancers often occur when there is too much estrogen in the body. These cancers are linked to changes in genes such as PTEN, PIK3CA, and those involved in repairing DNA mistakes, and they typically have a better chance of being treated successfully^[^18^]^. On the other hand, non-endometrioid cancers, such as serous, clear-cell, and carcinosarcoma, are more dangerous. They often have changes in the TP53 gene, which makes the cancer cells unstable and grow quickly. These types of cancers often occur when the lining of the uterus is thin^[^19^]^.

USC is a key example of Type II cancer. It resembles a type of aggressive ovarian cancer^[^20^]^. Women who have mutations in the BRCA1 or BRCA2 genes are more likely to develop serous or “serous-like” endometrial cancers instead of the usual endometrioid type. For example, a significant study found that the risk of developing serous-like endometrial cancer was much higher in women with BRCA1 or BRCA2 mutations compared to the general population^[^12^]^. Women with BRCA1 mutations had an even higher risk of developing serous-pattern endometrial cancer^[^3,12^]^. These cancers are often associated with problems involving the p53 gene, loss of the normal BRCA gene, and signs of poor DNA repair, which are similar to what is observed in ovarian cancers linked to BRCA mutations^[^3,21^]^. In fact, about 2–5% of women with USC have inherited BRCA1 mutations, which is higher than expected in the general population^[^3^]^. This suggests a possible connection between these mutations and the development of these cancers.

However, not all studies agree. Some studies, especially those conducted in European populations, did not find a significant increase in the risk of serous or all types of endometrial cancer in women with BRCA mutations^[^5^]^. Therefore, while the current evidence suggests that BRCA1 and BRCA2 mutations may increase the risk of developing aggressive, serous-type uterine cancers similar to those associated with BRCA-related ovarian cancers, further research is needed to understand why some studies yield different results^[^5,12,21^]^ (Table 1).

**Table 1:** The association between BRCA1/2 and the molecular and histopathological characteristics of uterine cancers.

Feature/pathway	Endometrioid (Type I)	Non-endometrioid/serous (Type II)	Clinical or therapeutic relevance
Common mutations	PTEN, PIK3CA, ARID1A, KRAS, CTNNB1^[^18^]^.	TP53, BRCA1, BRCA2, FBXW7^[^19^]^.	Differentiates aggressive, TP53-driven Type II tumors from low-grade, estrogen-related Type I^[^19^]^.
Hormonal background	Obesity and estrogen dependence are linked to each other^[^3^]^.	Atrophic endometrium; estrogen-independent^[^3^]^.	Impacts preventative measures and risk factors^[^3^]^.
BRCA1/2 alterations	Rare; typically inadvertent or somatic^[^12^]^.	In 2–5% of USC cases, germline or somatic BRCA1 deletion is seen^[^3^]^.	Indicates a similar pathogenic route to ovarian cancer linked to BRCA; it may also predict the sensitivity to PARP inhibitors^[^5,12^]^.
TP53 mutations	Infrequently^[^3^]^.	Worldwide (almost 90% of USC)^[^4^]^.	Determinant of poor prognosis and genomic instability^[^7^]^.
DNA repair phenotype	Commonly occurring mismatch repair deficiencies (MLH1, MSH2, PMS2, MSH6)^[^4^]^.	BRCA1/2-associated homologous recombination deficit (HRD) or associated gene loss^[^7^]^.	PARP inhibitors may be useful for HRD-positive tumors^[^12^]^.
P53 immunostaining pattern	Excessive focal expression or wild-type^[^3^]^.	Null pattern and widespread overexpression are examples of aberrant^[^3^]^.	Diagnostic indicator that separates Type I and Type II EC^[^18^]^
Histological pattern	Clearly distinct glandular^[^3^]^	Papillary, firm, superior, with noticeable atypia^[^3^]^	Directs the prognosis and diagnosis^[^12^]^
Tumor behavior	Slow, localized, and successful^[^3^]^	Aggressive, quickly spreading, and having a bad prognosis^[^3^]^	Impacts adjuvant treatment and the degree of surgery^[^7^]^.
BRCA-associated histotype	Rare; morphology similar to an endometrioid^[^5^]^	Serous-like or serous endometrial carcinoma^[^12^]^	Explains the overlap between ovarian and uterine serous carcinomas connected to BRCA1^[^3,12^]^.

### Clinical and genetic screening implications

In talking with people who have a BRCA1 or BRCA2 gene mutation, genetic counselors need to explain the uncertain risk of uterine cancer, especially for rare types like serous cancer, but also mention that the actual chance is still low^[^22^]^. They should consider other factors that might change a person’s risk, such as using tamoxifen, being overweight, or having a family history of endometrial cancer^[^22,23^]^. When thinking about preventive steps, they should work with the person to make a decision that fits their needs and values. Major guidelines, like those from NCCN and European experts, do not recommend regular screening for endometrial cancer, such as transvaginal ultrasound or endometrial biopsy, solely because someone has a BRCA mutation^[^24,25^]^. This is because there is not enough evidence that it helps, and there could be risks like false positives or unnecessary treatments^[^24^]^. However, doctors should be ready to check for unusual vaginal bleeding or spotting in BRCA carriers, especially if there are other concerns^[^25^]^.

For women who have their ovaries and fallopian tubes removed to lower cancer risk, deciding whether to also remove the uterus is tricky^[^26–28^]^. Some believe that removing the uterus means they can take estrogen-only hormone therapy, which avoids the need for progestin and might lower the small risk of serous endometrial cancer^[^23,29–31^]^. It might also make it easier to monitor their health. But others say removing the uterus does not provide much benefit, adds more risks during surgery, and there is not enough strong evidence that it improves survival^[^5,9^]^. Recent studies and reviews suggest that removing the uterus just for cancer prevention is not usually necessary for BRCA carriers^[^22,23,29^]^. Some experts suggest only considering uterus removal for BRCA1 carriers who are older or have other risk factors^[^22^]^.

When it comes to hormone replacement therapy (HRT), women who have their ovaries removed before menopause often need estrogen to help with menopause symptoms. If the uterus is still present, they also need progestin to prevent the lining of the uterus from growing too thick, which could lead to cancer. However, progestin might increase the risk of breast cancer in those with a genetic risk. If the uterus is removed, they can safely take estrogen-only therapy, which is less likely to increase breast cancer risk^[^30,31^]^. Other factors, such as being overweight, insulin resistance, using hormones, or taking tamoxifen, should also be considered when assessing cancer risk^[^22,23^]^. Ultimately, because there are not enough long-term data on cancer risk in BRCA carriers, any decisions about screening or surgery should be made on a personal level, based on genetic counseling, what the person wants, and carefully weighing the risks and benefits ^[^22–25^]^ (Table 2).

**Table 2:** Effects of brca1/2 mutation carriers on clinical and genetic counseling for uterine cancer.

Clinical consideration	Evidence/key findings	Clinical implication
Screening for endometrial cancer	Routine endometrial biopsies and transvaginal ultrasonography are not beneficial for BRCA carriers^[^24^]^.	Regular screening is not advised; only assess if there are symptoms^[^24,25^]^.
Tamoxifen use	For BRCA1 users, tamoxifen raises the SIR for endometrial cancer by up to 4.14.^[^4,23^]^	Talk about the hazards while administering tamoxifen, and if possible, take into account other options^[^4,23^]^.
Risk-reducing hysterectomy	Occasionally advised; however, older BRCA1 carriers or those with higher risks may want to explore it^[^22,23,26–29^]^.	Tailored strategy depending on the patient’s preferences, age, and risk factors^[^22,23,26–29^]^.
Hormone replacement therapy (HRT)	When coupled with estrogen, HRT may increase the risk of breast cancer^[^30,31^]^.	HRT in combination with the uterus is intact; estrogen alone if it is removed^[^30,31^]^.
Genetic counseling	Although the risk of uterine cancer is still minimal, serous subtypes have a greater risk^[^22,23^]^.	Provide patients with individualized advice based on their personal and family history regarding unknown but potential risk increases^[^22,23^]^.
Research and evidence gaps	Mixed cohort findings and a dearth of multiethnic, long-term data^[^6,12^]^.	Future studies ought to concentrate on sizable, potentially varied populations^[^6,12^]^.

### Challenges, confounding factors, and research gaps

Tamoxifen and HRT have complicated effects on the incidence of endometrial cancer among carriers of BRCA1 and BRCA2 mutations. The SIR for women who did not receive tamoxifen was 1.67 (95% CI: 0.81–3.07), while the SIR for those who did was 4.14 (95% CI: 1.92–7.87)^[^4^]^. Since the information that is currently available suggests that HRT is safe for young carriers who have undergone bilateral salpingo-oophorectomy, the concern about an elevated risk of breast cancer among BRCA carriers on HRT does not seem to be very significant ^[^32^]^. Despite hysterectomy’s effective prevention of endometrial cancer, a thorough risk–benefit analysis is necessary, informed by precise incidence estimates among women who are genetically predisposed. Nonetheless, hysterectomy is also associated with increased perioperative morbidity and longer hospital stays^[^4^]^.

The results of earlier cohort studies examining the risk of endometrial cancer in carriers of BRCA1 and BRCA2 mutations have been mixed, frequently as a result of small sample sizes, younger mean ages at recruitment, brief follow-up periods, or inadequate outcome validation^[^33^]^. Person-years at risk were computed from the date of the BRCA1/2 DNA test in order to reduce testing bias, as BRCA1/2 testing may occur after an endometrial cancer diagnosis^[^12^]^. When discussing prophylactic oophorectomy, gynecologists and patients benefit from an accurate assessment of endometrial cancer risk in mutation carriers^[^4^]^. This risk among carriers of BRCA1 and BRCA2 mutations was further assessed in a sizeable international prospective study^[^33^]^. Because of the small number of studies and their varied designs, results should be interpreted cautiously, even though meta-analyses show a statistically significant increase in endometrial cancer risk among carriers of the BRCA1/2 mutation. For patient counseling and treatment planning, accurate risk assessment remains essential ^[^6,12^]^.

Using multi-omics techniques to integrate genomic, transcriptomic, proteomic, metabolomic, and epigenomic data offers a thorough understanding of the molecular changes that underlie endometrial cancer. Finding sensitive and specific biomarkers for early diagnosis, prognosis prediction, and targeted therapy is made easier by this combination. According to recent research, TIMM8A and S100A2 may serve as prognostic indicators for patients with endometrial cancer. To maximize outcomes and guide future therapeutic efforts, molecular features must be incorporated into individualized treatment plans^[^33,34^]^.

### Clinical management and future directions

It is crucial to thoroughly assess the advantages and disadvantages of preventive measures. To guide management choices, accurate estimates of the incidence of endometrial cancer in women who are genetically predisposed to ovarian and breast malignancies are essential. Hysterectomy carries higher perioperative risks and a longer recovery period after surgery, despite its successful prevention of uterine cancer. Therefore, it is essential to accurately estimate the risk of endometrial cancer in carriers of BRCA mutations, as this knowledge helps patients and clinicians have conversations about prophylactic oophorectomy^[^4,6^]^. Except for patients receiving tamoxifen medication, there is currently no evidence to justify routine preventive hysterectomy for carriers of BRCA mutations^[^33–35^]^. Clinicians must determine who is at high risk and advise them of the benefits and drawbacks of different risk-reduction strategies^[^4,6,35^]^.

It is becoming more crucial to include molecular and genetic traits in customized therapy planning. The only proven preventive strategy for BRCA1/2 carriers at increased risk of ovarian cancer is risk-reducing surgery^[^12^]^. Emerging data suggest that PARP inhibitors can be used in conjunction with other targeted therapies to treat endometrial cancer, as they have shown encouraging efficacy in tumors with impaired DNA repair systems^[^36^]^. More accurate preventive and treatment approaches could result from a better knowledge of the molecular pathways causing BRCA-associated tumors^[^5^]^.

For BRCA mutation carriers, international guidelines, such as those issued by the European Society for Medical Oncology (ESMO) and the National Comprehensive Cancer Network (NCCN), place a growing emphasis on shared decision-making and individualized risk assessment. Standardizing care, while taking patient-specific genetic risk and reproductive objectives into consideration, can be achieved by incorporating molecular data, surgical timing, and pharmacologic prevention into these frameworks. Large, multiethnic, longitudinal cohort studies should be the main focus of future research in order to elucidate the lifelong risk of endometrial cancer in carriers of BRCA mutations^[^6,12,37^]^. The impact of hormone replacement treatment on the risk of uterine cancer in this population needs to be further studied. In order to improve risk prediction models and guide the creation of tailored clinical recommendations, it will be crucial to combine precision oncology techniques with molecular profiling.

## Conclusion

Genetic breast and ovarian cancers are tied to BRCA1 and BRCA2 mutations. Yet, more evidence shows that these genes might also impact uterine cancer, mainly the severe serous type. Even though the overall risk remains small, individuals with BRCA1 may face a slight yet significant increase in risk. Factors like having had breast cancer before and using tamoxifen make it worse. The need to think about usual changes, like TP53 changes and problems in cell repair, when we look at risk and ways to treat, is shown by the deep link between BRCA-related ovarian and uterine cancers. Because of different data and mix-ups caused by study limitations and group changes, the current guidelines remain cautious. However, we must prioritize closer monitoring in clinical settings, make collaborative decisions regarding preventive surgeries, and provide personalized advice based on an individual’s genetic profile. To better understand the risks and improve treatment approaches, future studies involving diverse populations over extended periods, as well as exploring tailored therapies, will be essential.

TITAN Guidelines: This manuscript is in compliance with the TITAN Guidelines, 2025, declaring no use of AI^[^38^]^.

## Data Availability

This narrative review is based on previously published literature. No new data were generated or analyzed in this study. Therefore, data sharing does not apply to this article.

## References

[1] Chebly A Yammine T Rassy E. Current evidence of BRCA mutations in genitourinary and gynecologic tumors: a scoping review. Precis Cancer Med 2022;5:17–17.

[2] Reitsma W Mourits MJE de Bock GH. Endometrium is not the primary site of origin of pelvic high-grade serous carcinoma in BRCA1 or BRCA2 mutation carriers. Mod Pathol 2013;26:572–78. 23080033 10.1038/modpathol.2012.169

[3] Pennington KP Walsh T Lee M. BRCA1, TP53, and CHEK2 germline mutations in uterine serous carcinoma. Cancer 2013;119:332–38. 22811390 10.1002/cncr.27720 PMC3966566

[4] Segev Y Iqbal J Lubinski J. The incidence of endometrial cancer in women with BRCA1 and BRCA2 mutations: an international prospective cohort study. Gynecol Oncol 2013;130:127–31. 23562522 10.1016/j.ygyno.2013.03.027

[5] Kitson SJ Bafligil C Ryan NAJ. BRCA1 and BRCA2 pathogenic variant carriers and endometrial cancer risk: a cohort study. Eur J Cancer 2020;136:169–75. 32698099 10.1016/j.ejca.2020.05.030 PMC7441309

[6] Zakerinasab F Behfar Q Parsaee R. BRCA 1/2 mutations and risk of uterine cancer: a systematic review and meta-analysis. BMC Genom Data 2024;25:13. 38297203 10.1186/s12863-024-01189-y PMC10829221

[7] Matanes E Volodarsky-perel A Eisenberg N. Endometrial cancer in germline brca mutation carriers: a systematic review and meta-analysis. J Minim Invasive Gynecol 2021;28:947–56. 33249269 10.1016/j.jmig.2020.11.023

[8] Roy R Chun J Powell SN. BRCA1 and BRCA2: different roles in a common pathway of genome protection. Nat Rev Cancer 2012;12:68–78. 10.1038/nrc3181 PMC4972490 22193408

[9] Zhao W Wiese C Kwon Y. The BRCA tumor suppressor network in chromosome damage repair by homologous recombination. Annu Rev Biochem 2019;88:221–45. 30917004 10.1146/annurev-biochem-013118-111058 PMC7004434

[10] Xu Z Xie H Song L. BRCA1 and BRCA2 in DNA damage and replication stress response: insights into their functions, mechanisms, and implications for cancer treatment. DNA Repair (Amst) 2025;150:103847. 40373656 10.1016/j.dnarep.2025.103847

[11] Petrucelli N Daly MB Pal T. BRCA1- and BRCA2-Associated Hereditary Breast and Ovarian Cancer. In: Adam MP Feldman J Mirzaa GM Pagon RA Wallace SE Amemiya A. eds.. GeneReviews®. Seattle:University of Washington, Seattle; 1993. 20301425

[12] De Jonge MM de Kroon CD Jenner DJ. Endometrial cancer risk in women with germline BRCA1 or BRCA2 mutations: multicenter cohort study. J Natl Cancer Inst 2021;113:1203–11. 33710348 10.1093/jnci/djab036 PMC8418438

[13] Ask D. Domchek: the Differences Between BRCA1 and BRCA2 | basser Center. Accessed 2025 Oct 22. https://www.basser.org/resources/ask-dr-domchek-differences-between-brca1-and-brca2

[14] Samstein RM Krishna C Ma X. Mutations in BRCA1 and BRCA2 differentially affect the tumor microenvironment and response to checkpoint blockade immunotherapy. Nat Cancer 2020;1:1188–203. 33834176 10.1038/s43018-020-00139-8 PMC8023400

[15] Mutations in BRCA1 or BRCA2 may increase risk for endometrial cancer. Mutations in BRCA1 or BRCA2 may increase risk for endometrial cancer. Accessed 2025 Oct 22. https://www.facingourrisk.org/XRAY/BRCA-mutations-linked-to-endometrial-cancer-risk

[16] Hall MJ Reid JE Burbidge LA. *BRCA1* and *BRCA2* mutations in women of different ethnicities undergoing testing for hereditary breast-ovarian cancer. Cancer 2009;115:2222–33. 19241424 10.1002/cncr.24200 PMC2771545

[17] Mefford HC Baumbach L Panguluri RCK. Evidence for a BRCA1 founder mutation in families of West African Ancestry. Am J Human Genet 1999;65:575–78. 10417303 10.1086/302511 PMC1377959

[18] Bokhman JV. Two pathogenetic types of endometrial carcinoma. Gynecol Oncol 1983;15:10–17. 6822361 10.1016/0090-8258(83)90111-7

[19] Hendrickson M Ross J Eifel P. Uterine papillary serous carcinoma: a highly malignant form of endometrial adenocarcinoma. Am J Surg Pathol 1982;6:93–108. 7102898 10.1097/00000478-198203000-00002

[20] Lee EK Fader AN Santin AD. Uterine serous carcinoma: molecular features, clinical management, and new and future therapies. Gynecol Oncol 2021;160:322–32. 33160694 10.1016/j.ygyno.2020.10.017

[21] Bogani G Ray-coquard I Concin N. Uterine serous carcinoma. Gynecol Oncol 2021;162:226–34. 33934848 10.1016/j.ygyno.2021.04.029 PMC9445918

[22] Domchek SM Friebel TM Singer CF. Association of risk-reducing surgery in BRCA1 or BRCA2 mutation carriers with cancer risk and mortality. JAMA 2010;304:967–75. 20810374 10.1001/jama.2010.1237 PMC2948529

[23] Kauff ND Satagopan JM Robson ME. Risk-reducing salpingo-oophorectomy in women with a BRCA1 or BRCA2 mutation. N Engl J Med 2002;346:1609–15. 12023992 10.1056/NEJMoa020119

[24] Rebbeck TR Lynch HT Neuhausen SL. Prophylactic oophorectomy in carriers of BRCA1 or BRCA2 mutations. N Engl J Med 2002;346:1616–22. 12023993 10.1056/NEJMoa012158

[25] Shu CA Pike MC Jotwani AR. Uterine cancer after risk-reducing salpingo-oophorectomy without hysterectomy in women with BRCA mutations. JAMA Oncol 2016;2:1434–40. 27367496 10.1001/jamaoncol.2016.1820 PMC5594920

[26] Nahshon C Segev Y Gemer O. Should the risk for uterine cancer influence decision making for prophylactic hysterectomy in BRCA1/2-mutated patients – a systematic review and meta-analysis. Gynecol Oncol 2021;160:755–62. 33309051 10.1016/j.ygyno.2020.11.034

[27] Gordhandas S Norquist BM Pennington KP. Hormone replacement therapy after risk-reducing salpingo-oophorectomy in patients with BRCA1 or BRCA2 mutations: a systematic review of risks and benefits. Gynecol Oncol 2019;153:192–200. 30661763 10.1016/j.ygyno.2018.12.014

[28] Huber D Seitz S Kast K. Hormone replacement therapy in BRCA mutation carriers and risk of ovarian, endometrial, and breast cancer: a systematic review. J Cancer Res Clin Oncol 2021;147:2035–45. 33885953 10.1007/s00432-021-03629-z PMC8164576

[29] Kotsopoulos J Lubinski J Huzarski T. Incidence of endometrial cancer in BRCA mutation carriers. Gynecol Oncol 2024;189:148–55. 39173195 10.1016/j.ygyno.2024.07.687

[30] Nair N Bowden J Bapat B. Hysterectomy at the time of risk-reducing surgery in BRCA mutation carriers: patient perspectives and clinical practice patterns. Fam Cancer 2018;17:405–10.

[31] NCCN. Treatment by cancer type. Accessed 2025 Oct 22. https://www.nccn.org/guidelines/category_1

[32] Birrer N Chinchilla C Del Carmen M. Is hormone replacement therapy safe in women with a BRCA mutation?: a systematic review of the contemporary literature. Am J Clin Oncol 2018;41:313–15. 26840041 10.1097/COC.0000000000000269

[33] Beiner ME Finch A Rosen B. The risk of endometrial cancer in women with BRCA1 and BRCA2 mutations. A prospective study. Gynecol Oncol 2007;104:7–10. 16962648 10.1016/j.ygyno.2006.08.004

[34] An Y Feng Q Jia L. Present progress in biomarker discovery of endometrial cancer by multi-omics approaches. Clin Proteom 2025;22:15. 10.1186/s12014-025-09528-6 PMC12032760 40281423

[35] Westin SN Broaddus RR. Personalized therapy in endometrial cancer: challenges and opportunities. Cancer Biol Ther 2012;13:1–13. 22198566 10.4161/cbt.13.1.18438 PMC3335980

[36] Musacchio L Caruso G Pisano C. PARP inhibitors in endometrial cancer: current status and perspectives. Cmar 2020;12:6123–35. 10.2147/CMAR.S221001 PMC7383016 32801862

[37] Guo SB Liu DY Fang XJ. Current concerns and future directions of large language model chatGPT in medicine: a machine-learning-driven global-scale bibliometric analysis. Int J Surg 2025;112:2805–22. 10.1097/JS9.0000000000003668 41133425

[38] Agha R Mathew G Rashid R. Transparency In The reporting of Artificial INtelligence – the TITAN guideline. Prem J Sci 2025;10:100082.

